# Strategies for public health adaptation to climate change in practice: social learning in the processionary Moth Knowledge Platform

**DOI:** 10.3389/fpubh.2023.1179129

**Published:** 2023-08-17

**Authors:** Yvette Buist, Marleen Bekker, Lenneke Vaandrager, Maria Koelen, Barbara van Mierlo

**Affiliations:** ^1^Department of Social Sciences, Health and Society, Wageningen University and Research, Wageningen, Netherlands; ^2^Department of Social Sciences, Knowledge Technology and Innovation, Wageningen University and Research, Wageningen, Netherlands

**Keywords:** public health adaptation to climate change, social learning, oak processionary moth, adaptation strategies, relationships, learning processes

## Abstract

Social learning theory can support understanding of how a group of diverse actors addresses complex challenges related to public health adaptation. This study focuses on one specific issue of public health adaptation: oak processionary moth (OPM) adaptation. With a social learning framework, we examined how public health adaption strategies gradually develop and are adjusted on the basis of new knowledge and experiences. For this qualitative case study, data were collected through 27 meetings of the Processionary Moth Knowledge Platform in the Netherlands and six additional interviews. Results indicate that relations between stakeholders, including experts played a major role in the learning process, facilitating the development and implementation of OPM adaptation and connecting local challenges to national adaptation strategies. Uncertainties regarding knowledge and organization were recurrent topics of discussion, highlighting the iterative and adaptive nature of public health adaptation. The study emphasizes the importance of building relationships among stakeholders and small steps in the learning process that can lead to the creation of new strategies and, if successful, the prevention of negative health impacts.

## 1. Introduction

Climate change has profound effects on human health, manifested through various pathways, such as altered rainfall patterns, extreme weather events, ocean acidification, and air pollution. Known health impacts of climate change include cardiovascular and respiratory risks, undernutrition, vector-borne diseases, as well as mental health issues (1–3). This study focuses on the (potential) climate change related health impacts of insect pests. Insect pests are influenced by both direct climatic factors and indirect factors, such as the length of the growing season, habitat structure, food quality, and overwintering (4–6). Increasing temperatures due to global warming may change the latitudinal distribution of insect pests. The multifaceted ecological mechanisms underlying insect pests responses to global warming increase the complexity of adapting to these changes (4, 7).

Public health adaptation is the process of designing, implementing, monitoring, and evaluating strategies, policies, and measures intended to adjust to actual or expected health effects caused by climate change (8, 9). Examples of public health adaptation include technical strategies (e.g., vector-elimination techniques, and data-driven disease surveillance platforms) and institutional strategies (e.g., expertise centers and networks, plans, and action aimed at preparing for severe weather) (10–13). In public health literature there is a growing recognition of the need for multisectoral and interdisciplinary responses to protect public health for the adverse impacts of climate change (14). In the current emerging literature pertaining to ecological approaches to health, for instance Planetary Health and OneHealth, multidisciplinarity is widely regarded as one of the fundamental cornerstones for establishing a connection between global health and sustainability (15, 16). These approaches advocate for the incorporation of multidisciplinary perspectives in global health research, education, program and policy evaluations, planning, and implementation, with the aim of connecting ongoing global health endeavors in disease control and preparedness (17, 18). However, putting these ideas into practice turns out to be quite difficult due to interdependencies between actors with differing perceptions of risks, competing values, and varying roles and responsibilities (10, 19, 20). Additional challenges to public health adaptation have to do with uncertainties concerning the pace of climate change, its effects on health systems, and the impact of adaptation strategies (21).

These challenges define the complex and dynamic character of public health adaptation; there is no blueprint for optimal public health adaptation strategies and multiple stakeholder groups have to share, integrate and create knowledge together (21, 22). For this reason, a social learning perspective may contribute to a flexible and adaptive approach to public health strategies. The development of effective public health adaptation among others involves continues learning to integrate new information regarding changing risks (23). If this happens, in practice actors would experiment with small-scale actions, learn along the way, and adapt their plans accordingly (24–26). Social learning theory provides a lens to analyse the processes through which actors develop a mutual understanding from scientific knowledge and practical experiences (27). By incorporating social learning into practices of health adaptation, stakeholders can foster collaborative relationships, develop a process of co-creation of knowledge and develop coordinated adaptation actions over time (28).

In this study, we focus on social learning in a platform in the context of a specific public health adaptation issue: adaptation to the oak processionary moth (OPM: *Thaumetopoea processionea*).^1^ The OPM has been identified as an emergent public health problem that is exacerbated by climate change, especially given its northward advance through Europe (29, 30). When humans come into contact with the poisonous hairs (setae) of the OPM, they often experience a reaction of the skin or mucous membranes, with some people experiencing conjunctivitis, pharyngitis, respiratory distress, and anaphylactic reactions (31, 32). Either directly or indirectly, OPM adaptation focuses on the prevention of disease caused by the OPM. In a previous study, we demonstrated that OPM adaptation is challenged by technical (e.g., limited resources), organizational (limited collaborations), and normative aspects (e.g., differing perceptions concerning health, sustainability and risk) (19). Given that the OPM is an endemic phenomenon, long-term OPM adaptation is needed (33).

This case study focuses on the role of social learning in the Dutch interdisciplinary Knowledge Platform (KP) on the processionary moth; a platform which was established to reduce or prevent the negative effects of OPM in 2019. We selected the KP as a case for studying multi- or interdisciplinary public health adaptation, as it is a newly established network in which a heterogeneous group of professionals frequently meets to address an emergent public health adaptation issue. The research question of this study is: “What is the role of social learning in developing OPM adaptation strategies in the KP?” The results shed light on influential events and the importance of relationships within a network, in addition to identifying ideas and experiences for the development of approaches to public health adaptation.

## 2. Analytical framework

Social learning theory can support understanding of how a group of diverse actors addresses complex adaptation challenges (34). It is often applied within the context of sustainability issues, as many of them have no clear-cut solutions and thus would benefit from learning processes aimed at concerted action (18). For example, Van Epp and Garside (35) applied a social learning approach the context of climate change and food insecurity in various African countries. This study suggests that engagement, capacity development, iterative learning, and challenging institutions contributed to the development outcomes in the context of climate change and food security challenges. Social learning theories emphasize the importance of including knowledge from multiple perspectives to develop approaches to complex problems. One precondition for social learning is therefore interdependency amongst a heterogeneous group of actors who are faced with a specific social-ecological issue (36). The outcomes of a social learning process are context-dependent. The decisions made in the social learning process are based on what is learned by whom and how this knowledge is put into action (37–39).

**Figure 1:**
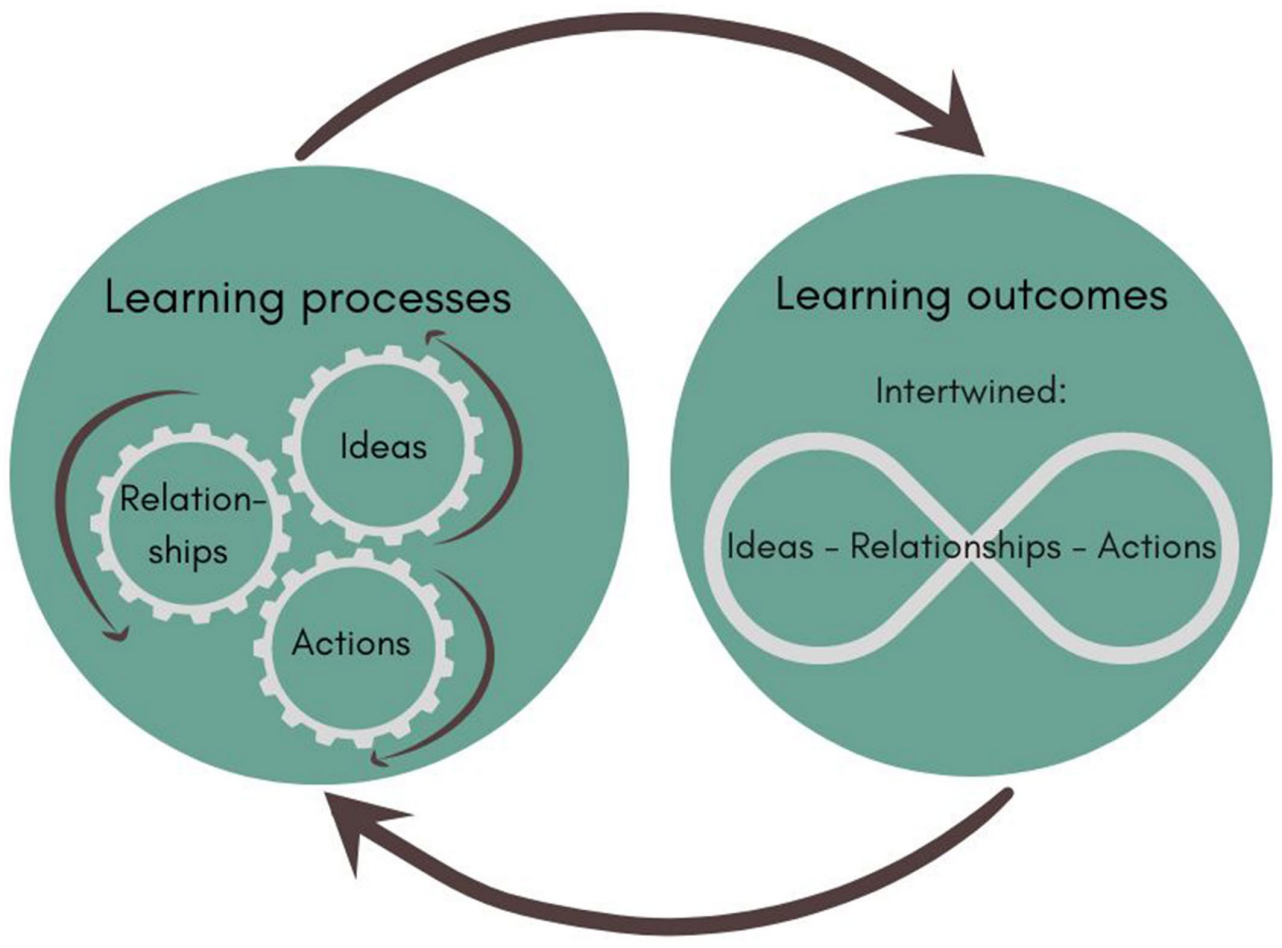
Learning process and outcomes, adapted from Beers et al. (37).

As noted in studies by Pahl-Wostl et al. (40) and Reed et al. (41) confusion often arises between the concept of social learning, the social learning process, and its potential outcomes. For this reason, the current study is based on the social learning framework developed by Beers et al. (37). This framework helps to unravel social learning processes, outcomes, and the ways in which learning might lead to concerned action within organized networks. In Figure 1, we show how the learning process is seen as an interactive process in which ideas (the “what”), actions (the “how”), and relationships (the “who”) change over time (37). These dimensions can be observed in the communication that takes place within networks. More specifically, ideas consist of new or changed ideas and perspectives, such as new knowledge, problem definitions and solutions, views and future visions, and (shared) values. Actions refer to new or modified actions, such as the development of new plans. Relationships consist of new and changed relationships and, therefore, dependencies between actors. They concern the involvement and efforts of a group of people who did not collaborate earlier. This learning process results in a learning outcome when ideas, actions and relationships become substantively interwoven and when they result in a decision, action, or reaction (37). Learning outcomes also serve as input for new learning processes. This can result in cycles in which learning processes contribute to learning outcomes, which subsequently contribute to new learning processes. In the current study, this social learning framework is applied to analyse public health adaptation to climate change in a specific case: the development of OPM adaptation strategies in the Netherlands.

## 3. Methods

As an emergent public health problem that is exacerbated by climate change, OPM is a suitable issue to study strategies for public health adaptation. The empirical focus of this article builds on a qualitative, participatory, in-depth case study of the Processionary Moth Knowledge Platform in the Netherlands. The first author monitored this initiative for a period of 29 months (April 2020–September 2022). In the following sections, we describe the contextual outline and case description, the study design, data collection, and methods of analysis.

### 3.1. Contextual outline and case description: the processionary moth knowledge platform

The OPM has been in the Netherlands since 1991. Local outbreaks have occurred once every 3–10 years, and local actors have tried to prevent the health impact of the OPM. Two strategies can be distinguished in these efforts to prevent the negative health impact of the OPM: measures focusing on ecological and chemical means and human-focused measures. Measures focusing on ecological and chemical means are aimed at monitoring and reducing OPM through various OPM management strategies, such as applying organic or chemical agents, reducing the number of oak trees, and increasing the presence of natural enemies by increasing biodiversity. Human-focused measures are aimed directly at humans and limiting their exposure to the OPM (e.g., informing citizens to avoid OPM or how to act when one comes into contact with OPM). These measures also include preventing or limiting OPM exposure for livestock and pets.

In 2012, the OPM Knowledge Centre (a different organization than the KP) was established by a group of researchers, biologists, medical environmental health specialists, and tree-maintenance specialists. They study the OPM and inform actors who are aiming to reduce or prevent the ecological and socio-economic effects of the OPM. Local actors (e.g., municipalities) have based their decisions on information from the OPM Knowledge Centre. Aside from the OPM Knowledge Centre, there has been little network collaboration to prevent the negative health impact of the OPM in the Netherlands. For a more in-depth discussion of OPM adaptation and the network of OPM actors, we refer to Buist et al. (19).

In 2019, the Netherlands experienced a major outbreak of OPM. Many people visited their general practitioners with health complaints. A wide range of actors—municipalities, citizens (including owners of pets and oak trees), general practitioners, community health services, sports associations, regional site-management organizations, maintenance contractors, and farmers—lacked sufficient knowledge and resources to prevent or reduce the negative health impact of the OPM. The outbreak also led to parliamentary questions, with parliamentarians calling for clear information on OPM adaptation. Issues relating to the OPM touch several policy domains—agriculture, nature, health, infrastructure, water management, and internal affairs—thereby complicating the commissioning process. The Ministry of Agriculture, Nature and Food Quality established the Processionary Moth Knowledge Platform in the summer of 2019. It was initially funded by three ministries (the Ministry of Agriculture, Nature and Food Quality; the Ministry of Health, Welfare and Sport; and the Ministry of Infrastructure and Water Management). The KP aimed to establish a uniform national information point for the prevention of health risks related to the OPM by collecting and communicating the latest insights about OPM adaptation and by supporting the development of OPM coordination structures.

The KP involves collaboration amongst various authorities (national, provincial, and local). Six working groups have been established: Monitoring; Control and Management; Health; OPM liability; OPM waste; and Communication. The KP collects and shares knowledge and information about these topics. The core group of the KP is composed of leaders from the working groups, representatives from several provinces, and the general secretary. This core group meets regularly (weekly or monthly during the main season). Every 6 months, a meeting is held with the steering committee, which includes representatives of the funding ministries, nature associations, regional medical support organizations, authorities (national, provincial, and local), the Interprovincial Consultation network, the Association of Dutch Municipalities, and various entrepreneurial trade organizations.

### 3.2. Study design and data collection

To collect data, participatory observation was employed. At the start of the study, the first author explained the purpose and method of the research and asked the KP members for permission to follow the KP over an extended period of time. Both the core group and the steering committee participants of the KP agreed to the researcher’s presence at the meetings and the use of the data for research purposes. Due to COVID-19, these meetings were held online. The first author acted as an observer-as-participant during the study (42, 43). This meant that the author mainly observed the meetings and participated in activities such analysing surveys conducted by the KP, creating an overview of stakeholders involved in OPM adaptation, and helping with inquiries regarding adaptation methods and procedures. This “peripheral membership role” enabled the researcher to observe and interact closely enough with the members of the KP to establish an insider’s identity without participating in activities that constitute the core of group membership (42). During these observations, the author made summaries of the meetings, including notes about new or changed ideas, actions, and relationships. Minutes and notes of 32 meetings were included for analysis, and the first author was present at 27 of these meetings.

The KP was established in the summer of 2019 and started operating in the spring of 2020. For this reason, 2020 was a good time to start the observation. Over the course of two and a half years, many activities were undertaken (e.g., establishment of new collaborations, dissemination of knowledge). The first author observed the meetings between 01-04-2020 and 01-10-2022. In addition to the observations, five semi-structured interviews were held with seven members of the core team in January 2021, with the goal of obtaining more detailed insight into the learning processes within the KP. All interviewees were informed about the purpose of the research prior to the interview and they were informed that participation is voluntary and they can withdraw their data from the study at any time. The interviewees have provided their consent for the usage of the interview content, under the condition that they were allowed to review a draft of the article and evaluate the accuracy of the information regarding the KP. A summary of the interviews was presented to the core group to allow them to gain an understanding of their learning process and to verify the summary. An additional interview was held with a policy advisor of the Ministry of Agriculture, Nature and Food Quality concerning the organization of OPM management. The analysis was based on a total of 80 documents, including minutes and notes (including verbatim transcriptions of insightful quotes) from the meetings of the core team and steering group, documents developed by the KP, and summaries of the six interviews (including the verbatim transcription of insightful quotes). The members of the steering committee and the core group were invited for a member check to assess the accuracy with which researchers had represented the data in the results, to enhance the quality of the data, and to serve as a fact-check (43).

### 3.3. Analysis

Qualitative content analyses was applied to all data, which provided a systematic way to make valid inferences from the data in order to describe learning in the KP (44). The research process described by Bengtsson (44), was used to analyse the data. The first phase was data familiarization by reading through the texts, to obtain a sense of what was going on. The themes of the framework developed by Beers et al. (37) were used during the process of coding and analyses, to generate insight into social learning within the KP and how it contributed to the development of adaptation strategies. The learning processes were viewed as a series of discursive interactions about ideas, relationships, and actions. Learning outcomes were established when ideas, actions, or relationships became intertwined and when they resulted in decisions or actions (37). These outcomes were arranged along a timeline. To ensure a structured approach and to generate insight into the interaction process, all entries describing ideas, actions, or relationships were coded (deductive coding) (Table 1). Ideas were coded for all statements describing new or changed ideas (i.e., which ideas were shared and which values were held). This included the state of affairs within the KP, as well as any problems, challenges, goals, visions, and strategies (e.g., discussions on which tasks the platform should perform). Next, new or changed actions describing how activities (should) take place were also coded. This included all expressions about the opportunity for action and proposed actions. Planned actions (e.g., such as a plan to search for more funding opportunities) were therefore included as well.

**Table 1:** Social learning processes within the KP.

What	Explanation	Examples
Ideas: new or changed ideas: which ideas were shared and which values were held	The development of ideas within the KP concerning its problems, challenges, goals, visions, and strategies	Discussions concerning the function that the platform should perform
Actions: new or changed actions: how activities (should) take place	All expressions about the opportunity for action and proposed actions. Actions could thus also include plans to act	An action plan for seeking more funding opportunities
Relationships: new or changed relationships: who is involved (describes the participation of actors)	Discussions of the role of internal or external stakeholders, interdependencies between actors, and the position of partners toward the KP	Changes in the composition of the partners involved in the platform

New or changed relationships concerned statements about the involvement and participation of actors. Relational utterances included discussions on the role of internal or external stakeholders, interdependencies between actors, and changes in the composition of the platform. Instances in which these processes resulted in decisions (i.e., when there was a clear connection between new or changed ideas, relationships, and actions, and when there was a plan to execute the action) were coded as learning outcomes. The researchers also paid attention to entries that did not fall into these categories, but which seemed relevant to the learning process and learning outcomes (inductive coding). The first author performed the deductive and inductive coding using the MaxQDA software. The coding was then double-checked with the second author. After the coding process, the recontextualization process took place. This involved checking whether all aspects of the content had been covered in relation to social learning and its contribution to the development of adaptation strategies. The data was re-read, and it was considered whether or not the unmarked text should be included. Then, the coded data was categorized, and themes were identified. These are the themes described in the results. The last stage was the compilation, where the coded data for each theme was revisited, and the writing process took place (44).

## 4. Results

### 4.1. Learning processes and outcomes related to key issues within the KP

**Figure 2:**
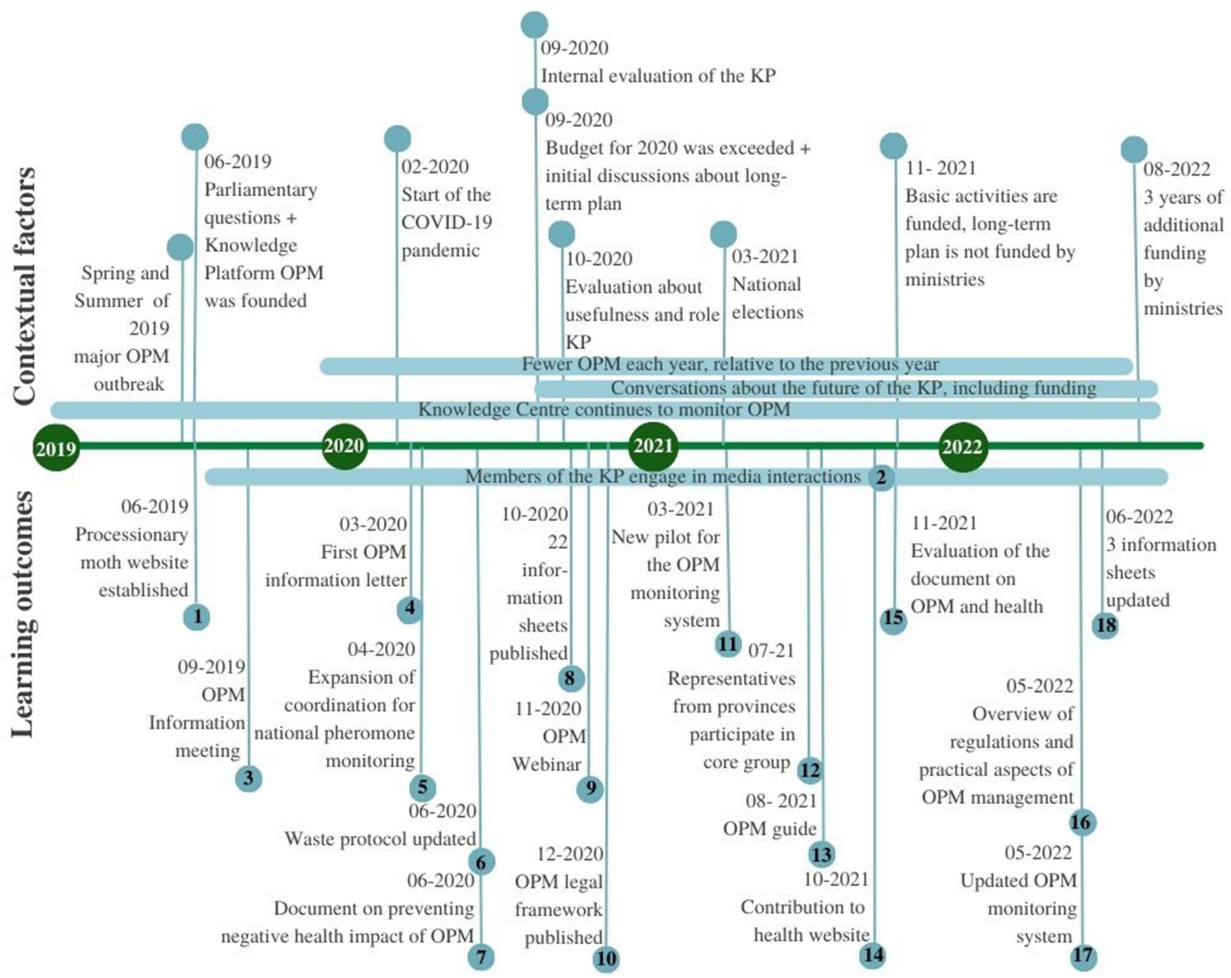
Timeline with contextual factors and outcomes in the learning process of the KP.

The analysis yielded a rich description of learning processes and outcomes within the KP, as well as external influential developments and events. In Figure 2, we present a timeline of the learning outcomes and important contextual factors. The upper part of the timeline displays events within the context of OPM adaptation that influenced the learning process. These events include political, and ecological factors, as well as factors unrelated to the OPM. Political factors include parliamentary questions, the mandate from the minister to establish a knowledge platform, and the funding of the KP. Ecological factors include the OPM outbreak and the reduction in the number of OPM. Unrelated factors include COVID-19 and the national elections. The learning process was also influenced by the internal and external evaluation of the KP and discussions on long-term planning. The lower part of the timeline displays the learning outcomes. Social learning outcomes are defined as moments when new or changed ideas, actions, and relationships became aligned in the communication of the platform. For example, the first outcome was the establishment of the processionary moth website.

The internal learning process taking place prior to the establishment of the processionary moth website (learning outcome) involved the KP agreeing that it would collect and share knowledge and information about the OPM (*idea*), several members of the KP building a website (*action*), and basing the content of the website on the input of various experts within and outside of the KP (*relationships*). The 18 learning outcomes identified in this study are presented in Table 2, substantiated by a description of the learning process.

**Table 2:** Learning processes and outcomes related to key issues within the KP.

Issue	Learning process	Learning outcomes
Lack of clear information about OPM adaptation for oak tree owners (e.g., municipalities, provinces)	Ideas: The role of the KP is to collect and share knowledge and information about the OPM	Outcome 1: Establishment of the processionary moth website (45) Outcome 2: Members of the KP engage in media interactions Outcome 3: OPM information meeting for municipalities and other interested parties Outcome 4: First OPM information letter for municipalities Outcome 9: OPM webinar for municipalities and other interested parties Outcome 13: OPM guide
	Actions: Developing the website, contacting media, answering questions (e.g., from citizens and municipalities), developing an information letter for municipalities, and organizing a webinar	
	Relationships: Various experts from the KP and other OPM experts provided information about various topics, including OPM management strategies, OPM monitoring, and OPM and health	
Uncertainty concerning what happens to OPM waste (OPM waste consists of nests and caterpillars removed from the trees)	Ideas: A safe method of waste disposal is necessary, as it is harmful to incinerate OPM waste along with regular waste	Outcome 6: Update to the OPM waste protocol
	Actions: Investigating what happens with OPM waste and developing an update to the OPM waste protocol	
	Relationships: Contact with waste companies for the transport and processing of OPM waste	
Uncertainty concerning the health impact of OPM and the influence of diverse management methods on human health	Ideas: A variety of factors determine the experienced health impact of the OPM; quantification of the health impact is challenging	Outcome 7: Document on preventing the negative health impact of OPM Outcome 15: Evaluation document on OPM and health
	Actions: Writing a document on preventing the negative health impact of OPM and performing an evaluation of OPM and health	
	Relationships: Experts from the KP and the Knowledge Centre collaborate in writing the documents	
Limited knowledge about OPM behavior and the predictability of pest pressure	Ideas: New methods for monitoring the OPM should be further developed	Outcome 5: Expansion of coordination for national pheromone monitoring Outcome 11: New pilot for the OPM monitoring system for municipalities and citizens Outcome 17: Updated OPM monitoring system for municipalities and citizens
	Actions: Developing and implementing monitoring systems for municipalities and volunteers to provide a quick and easy estimate of pest pressure	
	Relationships: KP, OPM Knowledge Centre, municipalities and volunteers collaborate on OPM monitoring	
Uncertainty about the effectiveness and potentially harmful side effects of OPM management methods	Ideas: (Dis)advantages of specific management methods, including steam and foil; addressing uncertainties and harmful and/or prohibited OPM management methods is necessary; KP should provide factual information on management methods	Outcome 8: 22 information sheets published Outcome 18: 3 information sheets updated
	Actions: Collecting existing knowledge concerning OPM management methods	
	Relationships: Collaboration with various experts on the information sheets	
Formal responsibilities of tree owners with regard to OPM are unclear	Ideas: Clarity is needed with regard to the legal implications for OPM adaptation	Outcome 10: The OPM legal framework published
	Actions: Developing an overview of legal frameworks and regulations	
	Relationships: Citizens and tourists send the KP questions concerning responsibilities, and the KP collaborated with a legal expert specialized in tree legislation	
Limited connection to regional authorities	Ideas: Connecting to regional authorities would increase insight into regional OPM challenges. Collaboration would increase the effectiveness of adaptation measures	Outcome 12: Expanding the core group with representatives from 3 provinces
	Actions: Instigating conversations with the Interprovincial Consultation network, the Association of Municipalities, and provinces	
	Relationships: The Interprovincial Consultation network participates in the steering committee. Three provinces considerably affected by the OPM were included in the core team of the KP	
How to distribute clear and comprehensive health information on OPM to citizens	Ideas: The website of the general practitioners is one of the most renowned health websites in the Netherlands	Outcome 14: Contribution of the KP to a renowned health website
	Actions: Providing additional health information for the website of the general practitioners	
	Relationships: Collaboration with general practitioners	
Unclear division of roles and responsibilities related to approving methods, and licensing	Ideas: No formal responsibility regarding the evaluation of management methods, but perceived responsibility to clarify roles	Outcome 16: Overview of regulations and practical aspects of OPM management
	Actions: Investigating the roles and responsibilities of various actors concerning the effectiveness and practical aspects of OPM management	
	Relationships: The KP interviewed stakeholders involved in the approval, licensing, and use of OPM management methods	

### 4.2. Contextual analysis of the role of social learning in the development of OPM adaptation strategies

The analysis revealed three themes concerning the role of social learning in the development of OPM adaptation strategies: (1) the importance of in-and external relationships in the development of OPM adaptation strategies; (2) the importance of relationships in the implementation of OPM adaptation strategies; and (3) revisiting the role and aim of the KP as part of the learning process. In this section, we describe how the learning processes led to the learning outcomes in relation to these themes. To illustrate the outcomes, we present several typical examples that provide the clearest representation of the learning process.

#### 4.2.1. Importance of in- and external relationships in the development of OPM adaptation strategies

The analyses indicated that relationships enhanced learning directed toward the development of adaptation strategies. The following is a description of relationships and the development of OPM adaptation strategies in connection to OPM information, and health, and the OPM.

At the onset, the KP received urgent questions from various parties (e.g., citizens, nature associations, and municipalities) about OPM adaptation strategies, the effectiveness and harmful side effects of these strategies, and the risks associated with them. To obtain information to answer these questions, the questions were discussed in the working groups and with experts outside the working groups (ideas and relationships). For example, to answer questions about safety for workers in the field controlling OPM, environmental health experts, the association of forest and nature owners, and contractors were asked for their views, and they were involved in formulating the answer. The KP shared this information on the website and also developed information emails and newsletters for municipalities (*action*) (Outcome 4). In addition, specific questions were received about OPM adaptation strategies. To formulate answers, the members of the Control and Management working group collaborated with a wide range of experts, including biologists, the Butterfly Conservation, developers of OPM control methods, and environmental health experts (*relationships*). These experts provided knowledge from their respective fields of expertise (*ideas*). Together, they developed 22 information sheets about common, promising, and less promising OPM management strategies for reducing the nuisance caused by the caterpillars (*action*) (Outcome 8).

“Additional [OPM] experts were approached by the working group and asked to contribute information [about adaptation strategies]. The fact sheets were created by approaching a shortlist of experts, who were asked for their input, ideas, and background information [on adaptation strategies]. Currently, there is a standardized questionnaire that is used when new methods are proposed to assess them.” (Data extract from actions list of the core group meeting)

With regard to health and the OPM, the exact health impact of the OPM is unclear, as is the influence of specific management strategies on the health of humans and animals (*idea*). Therefore, the prevention of the harmful OPM impact was investigated and OPM and health issues were evaluated (*action*). Various environmental health specialists, health researchers, ophthalmologists, and the association of general practitioners provided data and knowledge (*relationships*). For example, they reported that the number of OPM health complaints in 2020 was less than it had been in 2019. In addition, it became increasingly clear that the actual health impact is determined by a variety of factors, such as the number of management strategies in place, the type of weather, and the perceptions of citizens about the OPM (*idea*). This information resulted in the publication of documents on preventing the harmful impact of OPM and an evaluation of OPM and health (Outcomes 7 and 15).

The results of the analysis thus reveal that internal and external relationships (e.g., with biologists, the butterfly foundation, developers of OPM control methods, and environmental health experts) played an important role in the learning processes and outcomes of the KP. The learning outcomes were achieved through the contribution of a wide range of experts.

#### 4.2.2. Importance of relationships in the implementation of OPM adaptation strategies

In addition to the importance of relationships to the development of OPM adaptation strategies, relationships were beneficial to learning directed toward implementation. In this section, we describe relationships and the implementation of OPM adaptation strategies in connection to health, OPM waste, OPM monitoring, OPM management strategies, and the composition of the KP.

The Health working group was looking for ways to distribute clear and comprehensive OPM health information to citizens. They concluded that the website of the general practitioners is one of the most renowned health websites in the Netherlands. In collaboration with the association of general practitioners (*relationships*), they agreed to edit and write information about OPM and health for this website (*action*) (Outcome 14). The relationship with the general practitioners thus contributed to the accessibility of OPM information for citizens.

A: “We had exploratory discussions with the organization of general practitioners [about including OPM information on their website].” […]

B: “That's a good idea. Just make sure that the communication is aligned.”

C: “That's a good idea. They have a big reach, and on average, people tend to look at their website first.”

D: “I am positive about the idea.”

E: “Go for it. I think it will drive more traffic to their website and provide reliable information.”

(Members of the KP during core group meeting)

At the onset of the KP, it was unclear what was being done with OPM waste. The Waste working group contacted several companies and discussed whether they were able to work with OPM waste, how the waste should be packaged, and possible strategies and alternatives for transporting and processing OPM waste safely (*relationships + ideas*). Agreements were established with several waste processing companies, and the working group updated the previously written OPM waste protocol to inform oak tree owners and contractors about the storage, transport, and processing of OPM waste (*action*) (Outcome 6). This outcome is illustrated by the following quotation:

“…regarding the waste of 2019, a solution is gradually in sight. I will contact the waste processing company. The [OPM] waste that is now being vacuumed will be processed according to the established waste protocol. This will be evaluated at the end of the season and adjusted if needed.” (Member of the KP during a meeting of the core group)

In addition to uncertainties about waste, the level of OPM pest pressure (i.e., the number of and size of OPM nests) was unclear. Estimates of OPM pest pressure could improve the preparedness of municipalities and other tree owners with regard to OPM. To this end, the OPM Knowledge Centre developed a monitoring system in partnership with the Monitoring working group (*idea*). Municipalities and volunteers could communicate in an online document about the number of oak trees in which the OPM was present (*relationships and action*). The analysis of these observations was shared on several websites to inform municipalities and other tree owners about expected pest pressure (Outcomes 11 and 17). The development and implementation of this OPM monitoring system benefited from the efforts of municipalities and volunteers.

While developing the information sheets and addressing OPM questions, it became apparent that the division of roles and responsibilities related to the regulation of OPM management strategies was unclear. In agreement with the funding ministries, the KP received an additional budget to investigate the role and responsibilities of the different actors concerning the regulation of OPM management strategies. The KP interviewed the actors involved to create an overview of roles and responsibilities regarding regulations (*action*). Amongst other findings, they found that procedures for approving new OPM adaptation strategies are time-consuming and costly, that several different laws are applicable to OPM management, and that resources for regulating these laws are limited (*ideas*). Resources for dealing with the inappropriate labeling of OPM management methods and ineffective OPM strategies were also limited, due to a lack of knowledge and capacity on the part of national and regional authorities. This might lead to damage to the ecosystem (albeit unintentional). The KP shared its findings with the stakeholders, including the ministries. The KP noted that this investigation enhanced the relationships and network connections (*relationships*), and some stakeholders became more active in advancing OPM regulations (e.g., by examining whether information on pesticide packaging is correct and conducting conversations on how to deal with methods that seem dangerous to users or the environment) (Outcome 16)

“In consultation with the Human Environment and Transport Inspectorate, it will be determined whether newly marketed pesticides meet the requirements for proper and complete labeling, as well as the rules for application of pesticides.” (Data extract from notes from a core group meeting)

To increase contact with regional authorities and to enhance understanding concerning the implementation issues encountered by local authorities, and three provinces that had been considerably affected by the OPM joined the KP core team (*relationships*). The representatives from these provinces contributed to the KP by sharing their challenges, experiences, and ideas relating to the OPM (*ideas and actions*) (Outcome 12). For example, they shared challenges with available OPM management methods that are unsafe and harmful to users. The involvement of the provinces in the meetings of the core group ensured that these challenges could immediately be passed on to the competent authorities, with which the KP was also in contact. As a result, challenges did not remain a purely local issue, but could be scaled up if necessary.

The results of the analysis thus indicate that the implementation of OPM adaptation was enhanced by relationships with various parties, including general practitioners, the OPM Knowledge Centre, companies that process OPM waste, municipalities, and provinces. The cooperation with the provinces allowed the KP to gain more insight into the conditions needed for local application and implementation of OPM adaptation strategies, thereby decreasing the gap between local challenges and national OPM adaptation strategies. In addition to relationships, the role and the aim of the KP influenced the learning processes as well. We discuss this further in the next section.

#### 4.2.3. The role and aim of the KP as part of the learning process

In addition to the development and implementation of OPM adaptation strategies, the role and aim of KP were a subject of the learning process.

The KP aimed to establish an information point to prevent OPM health risks by collecting and communicating the latest insights about OPM adaptation and by supporting the development of OPM coordination structures. This aim was discussed repeatedly (e.g., with regard to uncertainties about the health impacts and effectiveness of such strategies). Despite the publication of documents health risks associated with the OPM and their prevention (Outcomes 7 and 15), some members noted that uncertainty remained with regard to the exact health impact of the OPM and the influence of different management strategies on these health risks for humans and animals. They preferred to have a stronger evidence base to show the urgency of OPM adaptation. Other members noted that, according to previous research, determining the exact impact is a complex process, regardless of additional efforts to define the impact. In addition, it was noted that the KP does not actually conducts empirical research, but creates overviews based on existing empirical data. No additional effort to conduct more research on the health impact of OPM on humans and animals was taking place. Given the lack of agreement on ideas regarding efforts to clarify the health impacts of the OPM, no action took place (≠ new learning process):

“The health effects must be made explicit, and this argument [health risks are a reason for intervention] must be stronger. What is required to move this [OPM adaptation] up the list of priorities?” (Member of the KP during a meeting of the core group)

Despite the development of 22 information sheets (Outcome 8), uncertainties also remained with regard to the effectiveness, harmful (or other) side effects, and risks associated with the various OPM management strategies.

The KP discussed its role and responsibility regarding these uncertainties. Within the core group, it was concluded that the KP aimed to help actors by providing them with information from OPM experts concerning OPM management strategies. It was agreed that the KP would provide only factual information about such strategies, and that the information would always include references. In light of remaining uncertainties, the KP was not able to answer all questions. Oak tree owners would have to make their own decisions and trade-offs between the beneficial and harmful effects of OPM adaptation strategies.

A: “From external actors, I heard there are rumours that we [the KP] are not really negative about current control methods”.

B: “The knowledge platform distinguishes fact from emotion. We have to be alert. We can explain the pros and cons of methods, but we do not take a position unless it is really harmful or proven to be ineffective (such as foil). Our communication needs to be clear, and the use of natural methods should be investigated. […] We must also acknowledge the adverse effects of current methods [e.g., of *bacillus thuringiensis*].” (Members of the KP during core group meeting)

At the onset of the KP, it was agreed that funding would be provided for 3 years, but that a plan would have to be approved by the funding ministries each year. In the summer of 2020, the KP core group and the municipalities conducted an evaluation of the KP. They were positive about the collaborations of the KP, the level of expertise, and the usefulness of the KP. At the same time, however, they noted the lack of a long-term vision and plan. Although the KP developed a project plan for the years 2021 and 2022, the ministries decided not to fund any newly proposed activities, due to the recent elections (which delayed decisions on budgets) and the priority assigned to COVID-19. Only primary activities (e.g., the organization of KP meetings and the evaluation of OPM and health) were funded (Outcome 15). Nevertheless, in several meetings of the steering committee, it was agreed that it was important to maintain a national OPM information network (e.g., a platform) to collect and share information, to prepare for the expected arrival of the pine processionary caterpillar, and to avoid the loss of existing knowledge and collaborations. This was a shared vision, despite the reduction in the number of OPM over the years 2020, 2021, and 2022. The core group and steering committee therefore considered which type of network would be most suitable for continuing long-term OPM adaptation. Examples considered included an integrated pest management network, a climate and health network, control of infectious diseases, and various ministerial programs (e.g., a green and healthy living environment). Although the ministries provided the KP with 3 years of additional funding in 2022, the future organization of OPM adaptation had yet to be determined at the time of this research.

“This is a network of exceptionally motivated people, and it would be a pity to lose the network and the knowledge. We can also use this as a case to learn from for similar problems that may arise in the future.” (Policy advisor at a ministry)

In light of persistent uncertainties, the best way to address them was a recurring point of discussion. The KP reconfirmed that they would not conduct empirical research. The role and aim of the platform itself were also an issue in the learning process, due to both uncertainties regarding effective public health adaptation and the contextual aspects of governmental support and conditions.

## 5. Discussion

In this study, we investigated how public health adaption strategies are gradually developed and adjusted in a social learning process. Adopting a social learning perspective toward public health adaptation, we examined how the iterative, adaptive process of learning within a network, in conjunction with its contextual factors, contributes to the interdisciplinary nature of this process. Our study revealed that the members of the KP developed their ideas on the present and future organization of OPM adaptation through an iterative process of cultivating relationships and arriving at novel insights and ideas, and subsequently acted upon them. Despite challenging contextual factors (national elections and COVID-19) and a reduction in the number of OPM in recent years, the KP was able to refine OPM adaptation strategies. At first, the KP focused mainly on addressing very concrete issues (e.g., OPM waste management and the dissemination of OPM management information). At the same time, the KP collaborated with various actors and expanded to include regional actors. The results indicate that these relationships played a major role in the learning processes and outcomes through the contributions of a wide range of experts. Relationships also enhanced the implementation of local OPM adaptation and drew links between local challenges and national OPM adaptation strategies. All activities of the KP took place in an iterative, adaptive process that could be characterized according to intermediate learning outcomes. Recurring issues in this process included the disclosure of available information about adaptation measures to municipalities, monitoring systems for defining and predicting pest pressure (Table 2), and the role and aims of the KP. Using the social learning framework allows for an in-depth understanding of how ideas, relationships, and actions collectively contributed to an overview of knowledge, regulations, and coordination between the organization of OPM adaptation at the national and provincial level.

This study suggests three external triggers for social learning processes that contribute to public health adaptation. One trigger may take the form of a request for information and/or action, as with parliamentary questions or questions from other parties (e.g., citizens, municipalities). These requests preceded the start of the KP. This demand is also mentioned by Füssel (46), in a study on adaptation planning for climate change. As described by Füssel, awareness of the problem is a prerequisite for learning within the context of adaptation. A trigger—often in the form of an extreme event—is needed for adaptation to arise (46). Awareness of the OPM was most likely also increased by the interest of the media in OPM and the engagement of OPM experts’ in media interactions. A second trigger has to do with funding. More specifically, the funding of the activities of the KP was a condition for the members of the KP to participate in the platform, and it was therefore a condition for actions related to public health adaptation. This trigger echoes the results of a study by Huang et al. (21), in which they also found that governmental prioritization, including funding mechanisms, is required to strengthen institutional capacity for timely public health adaptation. Third, given the emergent character of issues relating to public health adaptation, it is important for actors to recognize the need for collaboration and to develop capacity for dealing with changes. In the case of OPM adaptation in the Netherlands, the need to organize collaboration amongst diverse actors in the KP was recognized. This finding is in line with the dimensions of adaptive governance explored by Folke et al. (47), who identify fostering diversity for reorganization as one of several critical triggers for coping with rapid change (47).

The OPM is one of the many public health adaptation issues. Although the health impact of the OPM might be relatively small compared to other public health adaptation issues, the study of the KP shows that OPM adaptation is clearly a manifestation of complex coordination, scales and mechanisms involved in climate health adaptation. Given that public health adaptation is often an uncertain and open-ended process, the case of the KP confirms that learning is of key importance to the organization of multisectoral collaboration by networks of interdependent actors who are connected by a similar issue (e.g., flooding in a watershed) to develop adaptive and effective strategies (48).

Public health adaptation strategies, including OPM adaptation strategies, are not fixed processes with tightly defined outcomes (21). The complexity of public health adaptation issues relates to a wide range of stakeholders, informational uncertainty (i.e., limitations regarding knowledge about further developments), as well as normative uncertainty (i.e., related to goals, actions, and acceptable risks) (19, 39). Informational uncertainty can be addressed by applying alternative strategies, such as a focus on social learning processes rather than on prediction and control (39, 48). Emphasizing social learning processes and incorporating enriches the approach to addressing normative uncertainty. The participation of a wide range of stakeholders in the decision processes allows stakeholders to identify priorities (e.g., instance increasing biodiversity) and acceptable risks (e.g., human exposure to the OPM) (39). As argued by Beers et al. (49), the negotiation of meaning is particularly supportive of learning, as it requires a diverse group of stakeholders to arrive at a common understanding of the problem at the onset of collaboration. Integrated and adaptive forms of governance for public health adaptation with a focus on social learning can therefore enhance public health adaptation.

A wide variety of professionals were involved in the KP. This made it a good case for studying the development of learning processes, as multisectoral involvement is a precondition for social learning to take place (36). The structure of the KP, including the regular meetings, ensured that everyone was informed about the various sub-topics. This also allowed the members of the KP to ask each other questions and exchange knowledge, thereby contributing to the joint search for solutions. It is still uncertain whether the social learning outcomes related to public health adaptation are specific to a knowledge platform, or whether similar outcomes would also occur in other types of networks. In addition, the learning outcomes of the KP are not solely the result of internal learning processes. They are also the results of a combination of contextual aspects and external activities. It should also be noted that this study was conducted after years of OPM groundwork by the OPM Knowledge Centre and other actors who did not receive any funding for their activities. It is thus also important to recognize that investments of time and relationships required for learning had already taken place before this study. By developing a timeline that includes both process and contractual aspects, and by conducting additional interviews, we aimed to develop a comprehensive overview of the learning processes.

Our study was based on a single case. The scope should be broadened in further research assessing learning in public health adaptation (e.g., within the context of the tiger mosquito or West Nile virus) by including multiple cases of developing OPM strategies. Studying the ways in which other countries approach public health adaptation could further promote learning about various OPM strategies (50). Finally, social learning is an approach that can be used to enhance learning by all interdependent actors within a specific context (26, 37). Future research on OPM adaptation might therefore benefit from integrating the perspectives of citizens and a wide diversity of stakeholders with regard to their willingness and capacity to adopt adaptation strategies and the articulation of citizen engagement in adaptation processes (51).

In conclusion, our study shows that, in the Netherlands, the development and implementation of public health adaptation strategies is an iterative and adaptive learning process. To utilize knowledge, relevant stakeholders must be actively involved in this collaborative learning process. Learning processes can enhance the connection between national public health adaptation knowledge and the implementation of public health adaptation strategies within the local context. Public health adaptation benefits from the consideration of small steps in the learning process that serve as input for new relationships, ideas, and actions. This supports the creation of new public health adaptation strategies and, if successful, the prevention of negative health impact.

## Data availability statement

The original contributions presented in the study are included in the article, further inquiries can be directed to the corresponding author.

## Ethics statement

Verbal consent was obtained at the beginning of the study, and any potentially identifiable quotes were carefully reviewed and approved by the participants before the publication of the study.

## Author contributions

YB collected data, preformed the formal analysis, and wrote the first draft of the manuscript. All authors contributed to the conceptualization, design of the study, manuscript revision, read, and approved the submitted version.

## Funding

This research was funded by the internal funds of the Health and Society chair group at Wageningen University and Research in the Netherlands.

## Conflict of interest

The authors declare that the research was conducted in the absence of any commercial or financial relationships that could be construed as a potential conflict of interest.

## Publisher’s note

All claims expressed in this article are solely those of the authors and do not necessarily represent those of their affiliated organizations, or those of the publisher, the editors and the reviewers. Any product that may be evaluated in this article, or claim that may be made by its manufacturer, is not guaranteed or endorsed by the publisher.

## Footnotes

^1^The oak processionary caterpillar is the larval stage of the oak processionary moth. We use OPM to refer to both the caterpillar and the moth.
